# Smoking and its treatment in addiction services: Clients’ and staff behaviour and attitudes

**DOI:** 10.1186/1472-6963-14-304

**Published:** 2014-07-14

**Authors:** Camilla Cookson, John Strang, Elena Ratschen, Gay Sutherland, Emily Finch, Ann McNeill

**Affiliations:** 1 National Addiction Centre, Institute of Psychiatry, King’s College London, Addiction Sciences Building, 4, Windsor Walk, Denmark Hill, London SE5 8AF, UK; 2 Division of Epidemiology & Public Health, UK Centre for Tobacco and Alcohol Studies, University of Nottingham, City Hospital, Nottingham NG5, UK; 3 UK Centre for Tobacco & Alcohol Studies, Nottingham, UK; 4 Addictions Clinical Academic Group and Consultant Addictions Psychiatrist, South London & Maudsley NHS Foundation Trust, London, UK

**Keywords:** Smoking, Substance misuse, Nicotine dependence treatment, NHS, Addictions, Staff, Alcohol, Heroin

## Abstract

**Background:**

High smoking prevalence has been observed among those misusing other substances. This study aimed to establish smoking behaviours and attitudes towards nicotine dependence treatment among clients and staff in substance abuse treatment settings.

**Methods:**

Cross-sectional questionnaire survey of staff and clients in a convenience sample of seven community and residential addiction services in, or with links to, Europe’s largest provider of mental health care, the South London and Maudsley NHS Foundation Trust. Survey items assessed smoking behaviour, motivation to quit, receipt of and attitudes towards nicotine dependence treatment.

**Results:**

Eighty five percent (n = 163) and 97% (n = 145) response rates of clients and staff were achieved. A high smoking prevalence was observed in clients (88%) and staff (45%); of current smokers, nearly all clients were daily smokers, while 42% of staff were occasional smokers. Despite 79% of clients who smoked expressing a desire to quit and 46% interested in receiving advice, only 15% had been offered support to stop smoking during their current treatment episode with 56% reported never having been offered support. Staff rated smoking treatment significantly less important than treatment of other substances (p < 0.001), and only 29% of staff thought it should be addressed early in a client’s primary addiction treatment, compared with 48% of clients.

**Conclusions:**

A large unmet clinical need is evident with a widespread failure to deliver smoking cessation interventions to an extraordinarily high prevalence population of smokers in addiction services. This is despite the majority of smokers reporting motivation to quit. Staff smoking and attitudes may be a contributory factor in these findings.

## Background

Smoking is the largest global cause of preventable death and disease [1]. Over 10 years ago, UK research highlighted that smoking prevalence in substance misusers was considerably higher than that in the general population: over 90% of clients in an inpatient drug and alcohol detoxification unit [2], and methadone maintenance clinics [3,4] were recorded as smokers, compared to 27% in the general population at that time [5].

Given the adverse consequences of smoking, it is likely that substance misusers experience a high level of tobacco related health problems. Indeed, it has been observed that people who had received inpatient treatment for alcohol dependence were more likely to die from a tobacco-related, rather than alcohol related, cause [6]. Similarly, in a cohort of over 400 people dependent on narcotics, smoking was significantly related to the likelihood of dying over a ten-year period [7]. Furthermore, research has highlighted a synergistic interaction between alcohol and smoking for cancer risk [8]. Contrary to the prevailing belief, smoking cessation does not appear to impact negatively on success of abstinence from other substances; rather a body of evidence suggests continued nicotine dependence may be a risk factor for relapse [9-12], although the evidence is not always consistent [13].

Evidence from the US from the early 2000s suggests that substance misusers who smoke are motivated to quit [14,15], although whether motivation to quit translates to a desire for support and action is not so clear: whilst three-quarters of a sample of 542 tobacco-using adults in substance abuse treatment in Colorado were considering tobacco cessation sometime in the future, only a third wanted help and even fewer thought smoking cessation should be addressed alongside their substance abuse treatment [16]. Additionally a recent paper found that just over half of methadone clients surveyed in Australia were ‘not seriously thinking about quitting’ [17]. Given substance misusers’ high degree of dependence [18] and concurrent addiction, it seems likely that this population would find it hard to stop without support.

US research suggests advice and support are not routinely available for substance misuse clients undergoing treatment. A minority (8%) of clients with alcohol dependency reported that alcohol treatment counsellors encouraged them to ‘quit smoking now’, one third told them ‘quit in the future’ and a notable 24% told them ‘do not quit’ [15]. American and Australian literature highlight that this seems to result from the common misunderstandings that clients do not want to quit smoking, are unable to quit and quit attempts will have a negative impact on treatment of other substance use, as well as a lack of staff knowledge and training [19-22]. Staff attitudes towards smoking cessation programmes appeared to be influenced by the amount of continuing education in nicotine addiction received [23] and their own smoking behaviour [22]. Staff smoking also has the potential to normalise the behaviour and portray it as a therapeutic event [24].

Since the millennium, the UK has implemented a number of tobacco control measures, such as a ban on tobacco advertising, the implementation of a national network of NHS Stop Smoking Services and the implementation of a national smoke-free policy [25,26]. It is therefore important to assess whether smoking in substance misuse settings has reduced over this period. In addition, recently published NICE guidance aims to support smoking cessation, temporary abstinence and smokefree policies in all secondary healthcare settings including drug and alcohol services [27], and identifying current smoking behaviour and attitudes will help to inform policies and practice to ensure conformity with this guidance in the future.

This study therefore examined the following research questions:

1. What is the smoking behaviour and motivation to quit of substance misusers and staff in addiction services?

2. What smoking cessation support is provided in addiction services?

3. What are client smokers’ and staff views on the appropriateness and feasibility of the provision and receipt of nicotine dependence treatment in the context of treatment for other addictions?

## Methods

### Study design

In 2013, short questionnaire surveys for clients and staff were carried out in a convenience sample from four community drug treatment services (with substantial opiate substitution treatment, OST), one inpatient detoxification unit, and two residential rehabilitation services in, or with links to, Europe’s largest provider of mental health care, the South London and Maudsley NHS Foundation Trust. Each of these services was visited by a researcher for one day, with the exception of an inpatient detoxification unit where due to limited numbers of clients on the ward at any one time, two visits were necessary. The questionnaire was self-completed or, for clients who had difficulty reading or writing, the questions were read aloud. Questionnaires were anonymous and once completed, sealed in an envelope and returned.

### Participants

All clients and staff present, or visiting, each service on the day of the survey visit were approached and those who gave verbal consent were surveyed. No exclusion criteria were used.

### Measures

Staff and client questionnaires were designed to assess smoking behaviour and attitudes relating to smoking cessation and harm reduction within the context of other addictions.

For clients, questions collected clinical and demographic information on age, ethnicity, gender, and main substance(s) for which they were receiving treatment/support. Smoking status (‘have you ever smoked’ (yes/no); if yes, ‘in the past year have you smoked’ (daily/occasionally/never)) was recorded, and Heaviness of Smoking Index (HSI) was obtained for daily smokers using information on number of cigarettes smoked per day (CPD) and time to the first cigarette of the day (TTF) [28]. Motivation to quit was ascertained using the Motivation To Stop Scale (MTSS, [29]) and interest in talking to someone about trying to reduce the harmfulness of their smoking was recorded. Those who were interested in receiving advice were further asked about interest in different treatment options. Clients were asked if they had ever received support to stop smoking by clinicians and whether they had received support in their current treatment episode. Attitudes towards nicotine dependence treatment were explored, including a question relating to when in their primary addiction treatment smoking should be addressed (options included early, late or after their primary addiction treatment), and a question asking them to rate on a 10 point scale (ranging from 1 = *not at all appropriate,* to 10 = *definitely appropriate*) how appropriate they thought it was to treat smoking as an addiction requiring treatment.

For the staff questionnaire, professional status and area of interest (drugs, alcohol and/or tobacco) were requested. Respondents were asked how important they would rate treatment of a number of substances a client may be using and when in a client’s primary addiction treatment smoking should be addressed (as in client questionnaire). Staff confidence in supporting clients who want to give up smoking was assessed on a 10 point scale ranging from 1 = *not at all confident,* to 10 = *extremely confident*). As in the client questionnaire, smoking behaviour, motivation to quit and interest in receiving advice was determined.

### Analysis

Completed questionnaires were coded, entered and analysed in SPPS version 20 for Windows. Descriptive statistics were used to obtain medians, interquartile range and proportions. Chi squared tests were used to determine relationships between categorical variables and Wilcoxon Signed Ranks Tests were used to establish significant differences between paired ordinal ratings. Due to small numbers in each of the seven motivational categories, for statistical analysis, these were collapsed into three categories: those who wanted to quit imminently (in the next month, three months or soon), those who wanted to quit but did not know when, and those that did not want to quit (the ‘I don’t know’ category was not included in statistical analysis). Current smoking behaviour was categorised using frequency of smoking and HSI into ‘occasional’ , ‘daily (low addiction)’ , ‘daily (moderate addiction)’ and ‘daily (high addiction)’ smokers. Statistical significance was taken as p < 0.05 for all outcomes.

### Ethics statement

This study was deemed by the Research Ethics Team at the Institute of Psychiatry to be a service evaluation and approved by the local South London & Maudsley NHS Foundation Trust Audit Committee in December 2012.

## Results

One hundred and sixty three clients and 145 staff completed the questionnaire, with a response rate of 85% (191 clients approached) and 97% respectively (150 staff approached). One hundred and sixteen clients and 101 staff were from 4 NHS community services, 36 clients and 30 staff were from 2 non-NHS residential services and 11 clients and 14 staff were from an NHS residential service. Missing/invalid data on all of the questions was less than 10% of the total sample (with the exception of staff motivation to quit, 15% missing data; staff TTF and HSI, 16%; and clients indicating interest in varenicline and bupropion; 19% and 17% respectively). Missing/invalid data were excluded from analysis on an item-by-item basis.

### Clients

Clients’ demographic and clinical characteristics are shown in Table 1(A).

**Table 1:** Demographic and clinical characteristics of respondents

**(A) Client characteristics**	**% (n)**
**Sex**	*Total n =156*
Female	31 (49)
Male	69 (107)
**Age (years)**	*Total n = 152*
20-29	12 (18)
30-39	30 (45)
40-49	36 (54)
50-59	18 (28)
60+	5 (7)
**Ethnicity**	*Total n = 163*
White	82 (134)
Non-White	18 (29)
**Substance Use**	*Total n =163*
Alcohol	16 (26)
Heroin/Opiates	37 (60)
Crack/Cocaine	3 (5)
Other	3 (5)
Multiple Substances	31 (51)
Did not specify	10 (16)
**(B) Staff characteristics**	**% (n)**
**Professional Status**	*Total n = 145*
Manager	6 (8)
Registered Nurse	17 (25)
Manager and Registered Nurse	2 (3)
Student Nurse	4 (6)
Clinical Psychologist & Consultant Clinical Psychologist	5 (7)
Trainee/Assistant Psychologist	4 (6)
Training & Non-training Grade Doctor	3 (5)
Consultant Psychiatrist/Physician	1 (2)
Healthcare Assistant	6 (8)
Substance misuse worker/practitioner/key worker	17 (25)
Trainee & Qualified Counsellor	6 (9)
Admin and Support	14 (20)
Clinical Other	11 (16)
Non-Clinical Other	3 (5)
**Area of interest**	*Total n = 145*
Drugs	15 (22)
Alcohol	3 (4)
Tobacco	3 (4)
Drugs and Alcohol	38 (55)
Tobacco and Alcohol	1 (2)
Tobacco and Drugs	0 (0)
Drugs, Alcohol and Tobacco	21 (31)
None	19 (27)

### Smoking behaviour and motivation to quit

Ninety four percent (n = 154; 95% CI 90–97) of clients had smoked at some time in their life and 88% (n = 144; 95% CI 82–92) were currently smoking. Smoking status, HSI and motivation to quit are shown in Table 2, column (A). There was a significant relationship between gender and smoking status (daily, occasional, ex-smoker, and never) (*x*^*2*^ = 8.9, df = 3, p = 0.030), with a higher proportion of males being daily (88%), occasional (2%) and ex-smokers (8%), compared to females (84%, 0% and 4% respectively) and a higher proportion of females never smokers (12%) compared to males (2%). Smoking prevalence was similar across the different substances used [data not shown]. Seventy five per cent of the daily smokers were classified as at least moderately addicted according to the HSI and 79% of clients expressed a desire to quit.

**Table 2:** Smoking behaviour and motivation to quit of clients and staff

	**(A) Clients % (n)**	**(B) Staff % (n)**
**Smoking status (Whole sample)**	*Total n = 163*	*Total n = 144*
Never	6 (9)	30 (43)
Ex-smoker	6 (10)	25 (36)
Occasional smoker	1 (2)	19 (27)
Daily smoker	87 (142)	26 (38)
**CPD (Daily smokers)**	*Total n = 140*	*Total n = 38*
1-10 cigarettes	32 (45)	55 (21)
11-20 cigarettes	53 (74)	37 (14)
21-30 cigarettes	10 (14)	5 (2)
31+ cigarettes	5 (7)	3 (1)
**TTF (Daily smokers)**	*Total n = 130*	*Total n =32*
≤5 minutes	52 (68)	19 (6)
6-30 minutes	34 (44)	22 (7)
31-60 minutes	9 (12)	19 (6)
61+ minutes	5 (6)	41(13)
**HSI (Daily smokers)**	*Total n =130*	*Total n = 32*
0-2 (low addiction)	25 (33)	66 (21)
3-4 (moderate addiction)	64 (83)	31 (10)
5-6 (high addiction)	11 (14)	3 (1)
**Motivation to quit (All smokers)**	*Total n = 140*	*Total n = 57*
*Want to quit imminently (in the next month, three months or soon)*	40 (56)	49 (28)
*Want to quit but don’t know when*	39 (55)	21 (12)
*Do not want to quit*	19 (27)	26 (15)
*Do not know*	1 (2)	4 (2)

Forty six percent (66) of clients were interested in talking to someone about trying to reduce the harmfulness of their smoking behaviour; a further 21% did not know. Of those interested, the most common request was for advice on replacing some cigarettes with other forms of nicotine delivery (87%), but advice on gradual reduction (77%) and abrupt cessation (53%) were also popular (Table 3). There was a significant relationship between clients’ motivational status and interest in advice (*x*^*2*^ = 32.9, df = 4, p < 0.001) with 64% of those who wanted to quit imminently (in the next month, three months or soon), 44% of those who wanted to quit but didn’t know when and only 11% of those who didn’t want to quit, interested in advice.

**Table 3:** Clients’ interest in different smoking cessation and harm reduction advice

**If you are interested in receiving advice, what sort of things would you be interested in hearing about? Advice on -**	**% Yes (n)**	**% Unsure (n)**	**% No (n)**	**% Don’t know enough (n)**
*Stopping smoking abruptly (total n = 72)*	53 (38)	19 (14)	28 (20)	
*Stopping smoking by gradually reducing the no. of cigarettes smoked (total n = 73)*	77 (56)	5 (4)	18 (13)	
*Replacing some cigarettes with other forms of nicotine delivery (total n = 76)*	87 (66)	5 (4)	8 (6)	
*NRT to help with withdrawals (total n = 77)*	60 (44)	12 (9)	8 (6)	14 (18)
*Bupropion (Zyban) to help with withdrawals (total n = 65)*	17 (11)	8 (5)	6 (4)	69 (45)
*Varenicline (Champix) to help with withdrawals (total n = 63)*	17 (11)	6 (4)	6 (4)	70 (44)

### Treatment provision

Fifty six percent (78) of clients had never received support to stop smoking by clinicians and only 15% (19) had received support to stop smoking by clinicians in their current treatment episode.

Significant differences were found between different settings: residential (24% offered support during current treatment episode) and community (11% offered support) settings (*x*^*2*^ = 3.91, df =1, p = 0.048) and between NHS (11% offered support) and non NHS (31% offered support) settings, (*x*^*2*^ = 6.80, df = 1, p = 0.009). As all the community settings were NHS we looked to see if there was a difference between the one NHS residential and two non-NHS residential settings; the difference was not significant (9% and 31% offered support respectively, *x*^*2*^ = 1.97, p = 0.160), although the sample size for the one NHS residential setting was small.

### Attitudes towards nicotine dependence treatment

Clients rated the appropriateness of treating smoking as an addiction requiring treatment a median of 10 rated on a 10-point scale (interquartile range, IQR = 3). Forty eight percent (64) of clients felt smoking should be addressed early in their primary addiction treatment, 28% (37) late in their primary addiction treatment and 22% (30) after their primary addiction treatment (2% (3) ticked multiple time points).

### Staff

Staffs’ demographic and clinical characteristics are shown in Table 1(B). A quarter expressed tobacco among their area of interest and the largest professional categories (constituting 36% overall) were nurses/nurse managers or substance misuse workers.

### Smoking behaviour and motivation to quit

Seventy percent (n = 102; 95% CI 62–77) of staff had smoked at some point in their life and 45% (n = 65; 95%CI 37–53) were current smokers with 42% (95% CI 30–64) of these being occasional smokers. Thirty four per cent of the daily smokers were classified as being at least moderately addicted according to the HSI and 70% of staff expressed a desire to quit (Table 2, column (B)). Smoking prevalence was similar across the different professions although as shown in Table 1(B), numbers in some categories were very small [data not shown].

Thirty one percent (19) of staff (a further 8% did not know) were interested in talking to someone about trying to reduce the harmfulness of their smoking behaviour. Similar to the clients, there was a significant relationship between staffs’ motivational status and interest in advice (*x*^*2*^ = 12.5, df = 4, p 0.014), with 39% of those who wanted to quit imminently (in the next month, three months or soon) and 41% of those who wanted to quit but didn’t know when, interested in advice and 7% of those who didn’t want to quit interested in advice.

### Attitudes towards nicotine dependence treatment

Treatment for smoking was rated by staff as significantly less important than treatment for other substances a client may also be using along side their primary addiction (Table 4).

**Table 4:** Rating of importance of treating different named substances

If a client was primarily in treatment for x*, on a scale from 1 to 10, how important would you rate treatment of the following substances that they may also be using:			
	Primary substance: *Alcohol (total n = 131)	Primary substance: *Heroin (total n = 132)	Primary substance: *Cannabis (total n = 133)
	**Median (IQR)**	**Median (IQR)**	**Median**
**Alcohol**		10 (1)	9 (2)
**Heroin**	10 (1)		10 (1)
**Cannabis**	7 (5)	7 (4)	
**Benzos**	10 (2)	10 (2)	9 (3)
**Smoking**	5 (5)	5 (5)	7 (5)

NB There were significant differences between smoking and all other substances’ ratings in each primary substance groups; Wilcoxon Signed Ranks Tests, p < 0.001; IQR = inter-quartile range.

*Primary drug treatment.

There was no difference in attitudes towards nicotine dependence treatment according to staff smoking status; mean (IQR) ratings were: daily smoker 5 (6); occasional smoker 5.5 (6); ex-smoker 5 (4); never smoker 6 (5).

Twenty nine percent (40) of staff felt smoking should be addressed early in a client’s primary addiction treatment, 30% (46) late and 33% (46) after (7% ticked multiple time points). The median rating of staff confidence in supporting clients who want to give up smoking was 7 (IQR = 4). When only including clinical staff (excluding admin and support), this did not change.

## Discussion

Markedly elevated smoking prevalence was observed amongst substance misusers (88%) and addictions staff (45%), compared to the general population (19%) [30]; smoking prevalence among clients was similar to that reported over 10 years ago. Staff and client smokers displayed different patterns of smoking with an unusually high prevalence of occasional smoking being observed among staff (compared to an occasional smoking prevalence of < 3% in the general population at the time [31]. Over three-quarters of clients who smoked expressed a desire to quit, 22% within the next three months. While nearly half of the clients who smoked were interested in talking to someone about reducing the harmfulness of their smoking behaviour, 56% had never received support to stop smoking by clinicians, and only 15% had received support in their current treatment episode. This demonstrates a clear unmet clinical need which needs to be urgently addressed.

Our study has a number of limitations. We were restricted to using a convenience sample hence our results derive from participation of the readily available range of agencies and those present on the days and times of the visits. Despite this, there is no reason to think that the extremely high smoking prevalence observed here would be atypical, especially as smoking prevalence in the general population in London is lower than that nationally [32]. Another limitation is that the accuracy of self-reported responses given by staff and clients could not be verified. Additionally, for the small proportion of clients and staff (15% and 3% respectively) who did not complete the questionnaire, they may have declined either because they did not think a questionnaire about smoking was applicable to them if they did not smoke, or, alternatively, if they did smoke, they may not have wanted to disclose information about their behaviour. However our very high response rates make any such biases unlikely. In addition, our study has provided an up-to-date snapshot of smoking and treatment provision in England across a range of community and residential addiction treatment settings using standardised questions to enable comparisons with general population data.

The elevated prevalence of smoking in substance misusers observed in our sample is consistent with surveys carried out over a decade ago in the UK [2-4]. The lack of change in smoking prevalence is in contrast with the falling rates in the general population and needs to be addressed. It would appear that the implementation of the comprehensive tobacco control strategy in England is having little impact on this population of smokers. The lack of change in smoking behaviour does not reflect a hardened group of smokers not interested in quitting. Nearly 80% of client smokers in our sample expressed a desire to quit compared to 62% in the general population [29]. Discrepancies between clients’ motivation to quit and current support provision were apparent, with only a minimal proportion of clients receiving support in their current treatment episode. This is consistent with US literature showing a motivated client group [14,15] who are not receiving adequate support for smoking cessation [15]. There was a significant difference in support provision across treatment settings with residential and non-NHS settings more likely to offer support; however even in these ‘optimal’ settings less than a third of clients had been offered support during their current treatment episode. Given the distribution of our sample across treatment settings, we were not able to accurately determine whether the residential versus community or non-NHS versus NHS factors were the key discriminator for treatment provision. Contrary to previous research finding that motivation to quit did not translate into a desire for advice and motivated smokers did not think smoking should be addressed early in treatment [16], in our sample motivation was associated with a desire for advice, and nearly half of substance misusers felt that smoking should be treated early in their primary addiction treatment. This is in contrast to staff for whom 29% felt that smoking should be treated early in primary addiction treatment, with over half (58%) thinking it should be postponed until late in, or after, their primary addiction treatment. Client acceptability of different types of advice and treatment options were mixed. While advice on how to gradually reduce the number of cigarettes smoked, and nicotine substitution, were more popular than advice on abrupt quitting, just over half were still interested in advice on the latter. Nearly two thirds of smokers did not know enough about varenicline or bupropion to indicate an interest, which is of concern given that they are proven effective treatments.

Given the literature showing staff smoking may influence their attitudes towards supporting clients to give up [22], the elevated rates of staff smoking is a concern both in terms of their own health and the impact it is having on their clients. Staff smoking has previously ranged in the literature from 14% to 40% [22], and our results push the upper boundaries of this, at 45%. This is over twice as high as the general population prevalence and well above that recorded in general health professionals: smoking prevalence in general practitioners was recorded at only 4% [33]. Additionally, we found that medical professionals appeared to have a similar prevalence of smoking to the other professional groups.

Of interest is the unusual pattern of smoking we observed in staff, with a high proportion of occasional smokers. It is unclear whether this response is somehow more socially desirable than reporting daily smoking or whether such a smoking pattern highlights an attempt to cut down or quit or is just characteristic of the professional group. This should be further explored using qualitative methods. Staff confidence in treating nicotine dependence varied greatly and there appeared to be no difference across different staff groups including admin and support staff. Staff also rated the importance of treating nicotine dependence significantly lower than other substances.

Recent NICE guidance [27] indicates that support for smoking cessation and harm reduction needs to be integrated into substance misusers’ standard care; all clients’ smoking status, motivation to quit and desire for advice should be recorded and acted upon. Additionally, the range of available behavioural and pharmacological support options for quitting smoking needs to be made apparent to clients and integrated into a clear clinical pathway. Staff smoking behaviour needs to be addressed explicitly, having two-fold beneficial consequences not only to their own health, but also the health of their clients’ through the provision of better role models and support. Like clients, a motivation to quit in staff was apparent; however the relatively low number of staff smokers indicating a desire to talk to someone about their smoking may indicate a reluctance to accept help. Training needs are apparent to provide information around the health consequences of smoking including its synergistic effects with other substances, as well as to increase staffs’ own confidence in supporting clients. Research is needed to establish whether delivery of interventions to this group produces standard observed benefits, or whether special, more tailored interventions are required, potentially alongside a greater understanding of drug use patterns [34]. Additionally staff attitudes and behaviours need re-assessment following the implementation of a training programme designed to address some of the barriers to and misconceptions about nicotine dependence treatment in substance misusers. The South London and Maudsley Trust is now implementing the relevant NICE guidance and introducing nicotine dependent treatment as part of all its treatment care pathways.

## Conclusions

To conclude, a large unmet clinical need is apparent. Smoking prevalence is elevated in both staff and clients in substance misuse settings. Despite the majority of clients expressing a desire to quit smoking, there appears to be a widespread failure to deliver smoking cessation support and interventions. Staff attitudes regarding nicotine dependence treatment’s importance, a potential lack of confidence and their own smoking behaviour may be inadvertently maintaining this smoking culture.

## Competing interests

GS has acted as a consultant to the manufacturers of nicotine replacement formulations, bupropion and varenicline and given lectures sponsored by them. All other authors have no competing interests.

## Authors’ contributions

AM and JS conceived of the study and together with CC, ER, GS and EF designed the study and questionnaires. CC carried out the fieldwork. CC and AM wrote the first draft of the manuscript and all other authors revised it critically for important intellectual content. AM is corresponding author. All authors read and approved the final manuscript.

## Pre-publication history

The pre-publication history for this paper can be accessed here:

http://www.biomedcentral.com/1472-6963/14/304/prepub
